# 4-Methyl-*N*-(2-methyl­phen­yl)benzamide

**DOI:** 10.1107/S1600536811018770

**Published:** 2011-05-25

**Authors:** Vinola Z. Rodrigues, Marek Fronc, B. Thimme Gowda, Jozef Kožíšek

**Affiliations:** a Department of Chemistry, Mangalore University, Mangalagangotri 574 199, Mangalore, India; b Institute of Physical Chemistry and Chemical Physics, Slovak University of Technology, Radlinského 9, SK-812 37 Bratislava, Slovak Republic

## Abstract

The asymmetric unit of the title compound, C_15_H_15_NO, contains two independent mol­ecules, which differ in the dihedral angle between the aromatic rings [48.98 (9) and 57.48 (8)°]. The methyl groups in *para* positions are disordered over two equally occupied positions. An intra­molecular N—H⋯O hydrogen bond occurs. The crystal structure is stabilized by inter­molecular N—H⋯O hydrogen bonds which link the mol­ecules into chains running along the *b* axis.

## Related literature

For the preparation of the title compound, see: Gowda *et al.* (2003 ▶). For our study of the effect of substituents on the structures and other aspects of *N*-(ar­yl)-amides, see: Bhat & Gowda (2000 ▶); Bowes *et al.* (2003 ▶); Gowda *et al.* (2008 ▶, 2009 ▶); Saeed *et al.* (2010 ▶).
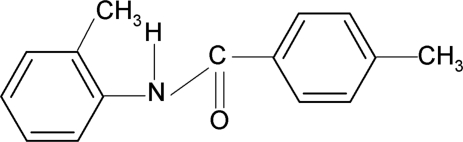


## Experimental

### 

#### Crystal data

C_15_H_15_NO
*M* *_r_* = 225.28
Triclinic, 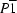

*a* = 7.2964 (6) Å
*b* = 9.9075 (5) Å
*c* = 18.1347 (13) Å
α = 88.331 (5)°
β = 82.892 (6)°
γ = 79.558 (5)°
*V* = 1279.29 (15) Å^3^
*Z* = 4
Mo *K*α radiation
μ = 0.07 mm^−1^
*T* = 293 K
0.88 × 0.09 × 0.06 mm

#### Data collection

Oxford Diffraction Xcalibur diffractometer with a Ruby (Gemini Cu) detector
Absorption correction: analytical [*CrysAlis RED* (Oxford Diffraction, 2009 ▶), based on expressions derived by Clark & Reid (1995 ▶)] *T*_min_ = 0.968, *T*_max_ = 0.996
18269 measured reflections
4354 independent reflections
1602 reflections with *I* > 2σ(*I*)
*R*_int_ = 0.088

#### Refinement

*R*[*F* ^2^ > 2σ(*F* ^2^)] = 0.042
*wR*(*F* ^2^) = 0.097
*S* = 0.74
4354 reflections
311 parameters
H-atom parameters constrained
Δρ_max_ = 0.12 e Å^−3^
Δρ_min_ = −0.11 e Å^−3^

### 

Data collection: *CrysAlis CCD* (Oxford Diffraction, 2009 ▶); cell refinement: *CrysAlis CCD*; data reduction: *CrysAlis RED* (Oxford Diffraction, 2009 ▶); program(s) used to solve structure: *SHELXS97* (Sheldrick, 2008 ▶); program(s) used to refine structure: *SHELXL97* (Sheldrick, 2008 ▶); molecular graphics: *ORTEP-3* (Farrugia, 1997 ▶) and *Mercury* (Macrae *et al.*, 2008 ▶); software used to prepare material for publication: *enCIFer* (Allen *et al.*, 2004 ▶).

## Supplementary Material

Crystal structure: contains datablocks I, global. DOI: 10.1107/S1600536811018770/bt5550sup1.cif

Structure factors: contains datablocks I. DOI: 10.1107/S1600536811018770/bt5550Isup2.hkl

Supplementary material file. DOI: 10.1107/S1600536811018770/bt5550Isup3.cml

Additional supplementary materials:  crystallographic information; 3D view; checkCIF report

## Figures and Tables

**Table 1:** Hydrogen-bond geometry (Å, °)

*D*—H⋯*A*	*D*—H	H⋯*A*	*D*⋯*A*	*D*—H⋯*A*
N1—H1*A*⋯O2	0.86	2.05	2.878 (2)	163
N2—H2*A*⋯O1^i^	0.86	2.05	2.883 (2)	162

Symmetry code: (i) 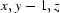.

**Fig. 1.**
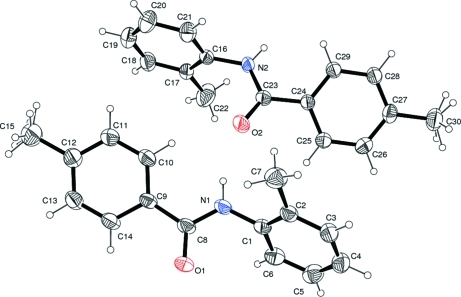
Molecular structure of the title compound showing the atom labelling scheme. Displacement ellipsoids are drawn at the 50% probability level. H atoms are represented as small spheres of arbitrary radii.

**Fig. 2.**
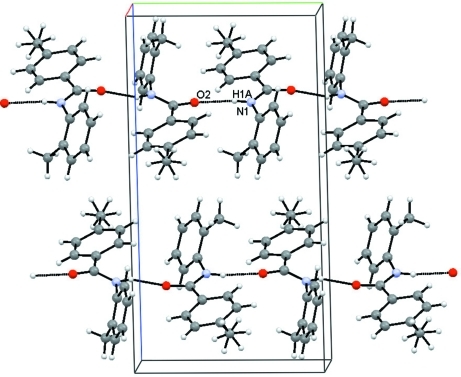
Part of the crystal structure of the title compound. Molecular chains are generated by N—H···O hydrogen bonds which are shown by dashed lines. H atoms not involved in intermolecular bonding have been omitted.

## supplementary crystallographic information

### Comment

The structural aspects of *N*-aryl amides are of interest due to their chemical and biological importance (Bhat & Gowda, 2000; Bowes *et al.*, 2003; Gowda *et al.*, 2008, 2009; Saeed *et al.*, 2010). In the present work, as part of a study of the substituent effects on the structures of benzanilides (Gowda, *et al.*, 2008, 2009), the structure of 4-methyl-*N*-(2-methylphenyl)benzamide (I) has been determined (Fig.1). The asymmetric unit of (I) contains two independent molecules. In the crystal, the *ortho*-methyl substituent in the anilino ring is positioned *syn* to the N–H bond in one of the molecules and *anti* in the other molecule. Further, the N—H and C=O bonds in the C—NH—C(O)—C segment are *anti* to each other in both the molecules, similar to that observed in 2-methyl-*N*-(4-methylphenyl)benzamide (II)(Gowda *et al.*, 2008) and 4-methyl-*N*-(2,6-dimethylphenyl)benzamide (III) (Gowda *et al.*, 2009) and, with similar bond parameters.

The central amide group –NHCO– is tilted to the anilino ring with the C2—C1—N1—C8 and C6—C1—N1—C8 torsion angles of -118.2 (3)° and 63.9 (3)° in molecule 1, and the C17—C16—N2—C23 and C21—C16—N2—C23 torsion angles of -86.8 (3)° and 97.6 (3)° in molecule 2, while the C10—C9—C8—N1 and C14—C9—C8—N1 torsion angles in molecule 1, and and the C25—C24—C23—N2 and C29—C24—C23—N2 torsion angles in molecule 2 are -13.4 (4)° and 171.7 (2)°, and -150.4 (2)° and 27.9 (4)°, respectively.

The packing of molecules linked by N—H···O hydrogen bonds into infinite chains is shown in Fig. 2.

### Experimental

The title compound was prepared according to the method described by Gowda *et al.* (2003). The purity of the compound was checked by determining its melting point. It was characterized by recording its infrared and NMR spectra. needle-like colourless single crystals of the title compound were obtained by slow evaporation from an ethanol solution of the compound (0.5 g in about 30 ml of ethanol) at room temperature.

### Refinement

All H atoms were visible in difference maps and then treated as riding atoms with C–H distances of 0.93Å (C-aromatic), 0.96Å (*C*-methyl) and N—H = 0.86 Å. The *U*_iso_(H) values were set at 1.2 *U*_eq_(C-aromatic, N) and 1.5 *U*_eq_(*C*-methyl). The methyl groups in p-position of the aromatic ring are disordered over two equally occupied positions rotated with respect to each other by 60°.

### Crystal data

**Table:**

C_15_H_15_NO	*Z* = 4
*M**_r_* = 225.28	*F*(000) = 480
Triclinic, *P*1	*D*_x_ = 1.170 Mg m^−^^3^
Hall symbol: -P 1	Mo *K*α radiation, λ = 0.71073 Å
*a* = 7.2964 (6) Å	Cell parameters from 2440 reflections
*b* = 9.9075 (5) Å	θ = 3.5–29.3°
*c* = 18.1347 (13) Å	µ = 0.07 mm^−^^1^
α = 88.331 (5)°	*T* = 293 K
β = 82.892 (6)°	Needle, colorless
γ = 79.558 (5)°	0.88 × 0.09 × 0.06 mm
*V* = 1279.29 (15) Å^3^	

### Data collection

**Table:**

Oxford Diffraction Xcalibur diffractometer with a Ruby (Gemini Cu) detector	4354 independent reflections
Radiation source: fine-focus sealed tube	1602 reflections with *I* > 2σ(*I*)
graphite	*R*_int_ = 0.088
ω scans	θ_max_ = 24.7°, θ_min_ = 4.1°
Absorption correction: analytical [*CrysAlis RED* (Oxford Diffraction, 2009), based on expressions derived by Clark & Reid (1995)]	*h* = −8→8
*T*_min_ = 0.968, *T*_max_ = 0.996	*k* = −11→11
18269 measured reflections	*l* = −21→21

### Refinement

**Table:**

Refinement on *F*^2^	Primary atom site location: structure-invariant direct methods
Least-squares matrix: full	Secondary atom site location: difference Fourier map
*R*[*F*^2^ > 2σ(*F*^2^)] = 0.042	Hydrogen site location: inferred from neighbouring sites
*wR*(*F*^2^) = 0.097	H-atom parameters constrained
*S* = 0.74	*w* = 1/[σ^2^(*F*_o_^2^) + (0.0367*P*)^2^] where *P* = (*F*_o_^2^ + 2*F*_c_^2^)/3
4354 reflections	(Δ/σ)_max_ < 0.001
311 parameters	Δρ_max_ = 0.12 e Å^−^^3^
0 restraints	Δρ_min_ = −0.11 e Å^−^^3^

### Special details

**Table:**

Experimental. CrysAlis RED (Oxford Diffraction, 2009) Analytical numeric absorption correction using a multifaceted crystal model based on expressions derived (Clark & Reid, 1995).
Geometry. All e.s.d.'s (except the e.s.d. in the dihedral angle between two l.s. planes) are estimated using the full covariance matrix. The cell e.s.d.'s are taken into account individually in the estimation of e.s.d.'s in distances, angles and torsion angles; correlations between e.s.d.'s in cell parameters are only used when they are defined by crystal symmetry. An approximate (isotropic) treatment of cell e.s.d.'s is used for estimating e.s.d.'s involving l.s. planes.
Refinement. Refinement of *F*^2^ against ALL reflections. The weighted *R*-factor *wR* and goodness of fit *S* are based on *F*^2^, conventional *R*-factors *R* are based on *F*, with *F* set to zero for negative *F*^2^. The threshold expression of *F*^2^ > σ(*F*^2^) is used only for calculating *R*-factors(gt) *etc*. and is not relevant to the choice of reflections for refinement. *R*-factors based on *F*^2^ are statistically about twice as large as those based on *F*, and *R*- factors based on ALL data will be even larger.

### Fractional atomic coordinates and isotropic or equivalent isotropic displacement parameters (Å^2^)

**Table:**

	*x*	*y*	*z*	*U*_iso_*/*U*_eq_	Occ. (<1)
O1	0.3561 (3)	0.83584 (15)	0.23183 (11)	0.0969 (7)	
N1	0.3243 (3)	0.62248 (17)	0.26640 (11)	0.0687 (7)	
H1A	0.3695	0.5366	0.2607	0.082*	
C1	0.1559 (5)	0.6596 (2)	0.31651 (18)	0.0614 (8)	
C2	0.1579 (5)	0.6225 (2)	0.39053 (18)	0.0622 (8)	
C3	−0.0084 (6)	0.6555 (3)	0.43740 (17)	0.0776 (9)	
H3A	−0.0099	0.6321	0.4875	0.093*	
C4	−0.1703 (5)	0.7216 (3)	0.4122 (2)	0.0857 (10)	
H4A	−0.2804	0.7421	0.4447	0.103*	
C5	−0.1687 (5)	0.7572 (3)	0.3388 (2)	0.0881 (10)	
H5A	−0.2783	0.8021	0.3212	0.106*	
C6	−0.0056 (6)	0.7270 (3)	0.29071 (17)	0.0768 (9)	
H6A	−0.0048	0.7522	0.2409	0.092*	
C7	0.3332 (5)	0.5461 (3)	0.41909 (16)	0.0928 (10)	
H7A	0.3634	0.4553	0.3987	0.139*	
H7B	0.3129	0.5403	0.4723	0.139*	
H7C	0.4352	0.5940	0.4044	0.139*	
C8	0.4170 (4)	0.7118 (2)	0.22826 (14)	0.0648 (8)	
C9	0.5994 (4)	0.6581 (2)	0.18350 (14)	0.0590 (7)	
C10	0.6996 (5)	0.5260 (2)	0.18947 (15)	0.0772 (9)	
H10A	0.6467	0.4619	0.2194	0.093*	
C11	0.8757 (5)	0.4888 (3)	0.15171 (16)	0.0832 (9)	
H11A	0.9396	0.3994	0.1565	0.100*	
C12	0.9607 (5)	0.5800 (3)	0.10686 (15)	0.0772 (9)	
C13	0.8592 (5)	0.7099 (3)	0.09924 (15)	0.0803 (10)	
H13A	0.9114	0.7729	0.0682	0.096*	
C14	0.6840 (5)	0.7481 (2)	0.13623 (15)	0.0736 (9)	
H14A	0.6192	0.8367	0.1297	0.088*	
C15	1.1607 (5)	0.5405 (3)	0.06924 (18)	0.1129 (12)	
H15A	1.2388	0.4932	0.1042	0.135*	0.50
H15B	1.2072	0.6218	0.0518	0.135*	0.50
H15C	1.1624	0.4816	0.0280	0.135*	0.50
H15D	1.2459	0.5820	0.0963	0.135*	0.50
H15E	1.1741	0.5717	0.0194	0.135*	0.50
H15F	1.2029	0.4415	0.0714	0.135*	0.50
O2	0.3997 (3)	0.32712 (14)	0.26391 (11)	0.0970 (7)	
N2	0.4584 (3)	0.10245 (17)	0.23914 (11)	0.0622 (6)	
H2A	0.4231	0.0247	0.2478	0.075*	
C16	0.6234 (4)	0.1063 (2)	0.18894 (17)	0.0541 (7)	
C17	0.6124 (4)	0.1340 (2)	0.11454 (19)	0.0665 (8)	
C18	0.7779 (6)	0.1290 (3)	0.06697 (17)	0.0799 (9)	
H18A	0.7723	0.1486	0.0168	0.096*	
C19	0.9474 (5)	0.0960 (3)	0.0923 (2)	0.0806 (9)	
H19A	1.0564	0.0931	0.0595	0.097*	
C20	0.9594 (5)	0.0668 (3)	0.1660 (2)	0.0866 (9)	
H20A	1.0757	0.0432	0.1834	0.104*	
C21	0.7952 (5)	0.0732 (2)	0.21414 (16)	0.0737 (9)	
H21A	0.8018	0.0546	0.2644	0.088*	
C22	0.4253 (5)	0.1644 (4)	0.08421 (19)	0.1311 (13)	
H22A	0.3592	0.0897	0.0962	0.197*	
H22B	0.4455	0.1755	0.0312	0.197*	
H22C	0.3524	0.2473	0.1060	0.197*	
C23	0.3531 (4)	0.2138 (2)	0.27375 (14)	0.0615 (8)	
C24	0.1817 (4)	0.1941 (2)	0.32233 (13)	0.0551 (7)	
C25	0.0317 (4)	0.3016 (2)	0.33069 (14)	0.0659 (8)	
H25A	0.0424	0.3857	0.3080	0.079*	
C26	−0.1335 (4)	0.2853 (2)	0.37224 (15)	0.0718 (8)	
H26A	−0.2340	0.3584	0.3766	0.086*	
C27	−0.1537 (4)	0.1625 (3)	0.40784 (14)	0.0667 (8)	
C28	−0.0014 (4)	0.0571 (2)	0.40096 (14)	0.0663 (8)	
H28A	−0.0101	−0.0255	0.4256	0.080*	
C29	0.1638 (4)	0.0716 (2)	0.35820 (13)	0.0618 (8)	
H29A	0.2639	−0.0017	0.3535	0.074*	
C30	−0.3368 (4)	0.1441 (3)	0.45296 (17)	0.1005 (10)	
H30A	−0.3499	0.1904	0.4996	0.121*	0.50
H30B	−0.3368	0.0481	0.4618	0.121*	0.50
H30C	−0.4397	0.1821	0.4260	0.121*	0.50
H30D	−0.3245	0.0952	0.4985	0.121*	0.50
H30E	−0.4078	0.0901	0.4238	0.121*	0.50
H30F	−0.4215	0.2321	0.4626	0.121*	0.50

### Atomic displacement parameters (Å^2^)

**Table:**

	*U*^11^	*U*^22^	*U*^33^	*U*^12^	*U*^13^	*U*^23^
O1	0.0967 (17)	0.0388 (9)	0.1473 (19)	−0.0146 (10)	0.0214 (13)	−0.0057 (10)
N1	0.092 (2)	0.0376 (11)	0.0721 (16)	−0.0128 (12)	0.0087 (15)	−0.0023 (11)
C1	0.072 (3)	0.0414 (14)	0.071 (2)	−0.0138 (16)	−0.001 (2)	−0.0075 (14)
C2	0.075 (3)	0.0501 (14)	0.062 (2)	−0.0146 (15)	−0.006 (2)	−0.0012 (14)
C3	0.085 (3)	0.0691 (18)	0.077 (2)	−0.0195 (18)	0.007 (2)	−0.0019 (16)
C4	0.079 (3)	0.078 (2)	0.099 (3)	−0.024 (2)	0.013 (2)	−0.0113 (19)
C5	0.067 (3)	0.082 (2)	0.117 (3)	−0.0108 (17)	−0.018 (3)	−0.012 (2)
C6	0.088 (3)	0.0662 (17)	0.078 (2)	−0.0120 (18)	−0.018 (2)	−0.0020 (16)
C7	0.097 (3)	0.0908 (19)	0.088 (2)	−0.0066 (19)	−0.018 (2)	0.0073 (16)
C8	0.084 (2)	0.0408 (14)	0.071 (2)	−0.0161 (16)	−0.0057 (17)	−0.0070 (14)
C9	0.080 (2)	0.0410 (14)	0.0574 (18)	−0.0154 (15)	−0.0075 (16)	−0.0022 (13)
C10	0.091 (3)	0.0512 (16)	0.086 (2)	−0.0196 (17)	0.008 (2)	0.0036 (14)
C11	0.092 (3)	0.0567 (16)	0.094 (2)	−0.0100 (17)	0.012 (2)	−0.0026 (16)
C12	0.090 (3)	0.0732 (19)	0.067 (2)	−0.0194 (19)	0.0062 (19)	−0.0126 (16)
C13	0.105 (3)	0.0621 (18)	0.071 (2)	−0.0251 (18)	0.014 (2)	−0.0009 (15)
C14	0.104 (3)	0.0491 (15)	0.066 (2)	−0.0159 (17)	0.0013 (19)	0.0037 (14)
C15	0.113 (3)	0.106 (2)	0.112 (3)	−0.019 (2)	0.019 (3)	−0.0098 (19)
O2	0.1053 (17)	0.0411 (9)	0.1356 (17)	−0.0257 (10)	0.0425 (13)	−0.0120 (10)
N2	0.0637 (17)	0.0417 (11)	0.0764 (16)	−0.0172 (11)	0.0228 (14)	−0.0052 (10)
C16	0.057 (2)	0.0420 (13)	0.063 (2)	−0.0137 (14)	0.0015 (19)	−0.0012 (13)
C17	0.052 (2)	0.0700 (16)	0.074 (2)	−0.0101 (14)	−0.001 (2)	0.0147 (15)
C18	0.075 (3)	0.093 (2)	0.071 (2)	−0.0203 (18)	0.000 (2)	0.0133 (15)
C19	0.070 (3)	0.094 (2)	0.078 (3)	−0.0254 (19)	0.009 (2)	−0.0146 (17)
C20	0.060 (3)	0.117 (2)	0.086 (3)	−0.0205 (18)	−0.012 (2)	−0.0166 (19)
C21	0.074 (3)	0.0844 (19)	0.064 (2)	−0.0216 (18)	−0.002 (2)	−0.0082 (15)
C22	0.074 (3)	0.192 (4)	0.116 (3)	0.002 (2)	−0.012 (3)	0.040 (2)
C23	0.069 (2)	0.0423 (14)	0.0704 (19)	−0.0121 (14)	0.0078 (16)	−0.0048 (13)
C24	0.063 (2)	0.0440 (14)	0.0565 (18)	−0.0142 (14)	0.0097 (15)	−0.0070 (12)
C25	0.068 (2)	0.0494 (15)	0.076 (2)	−0.0110 (16)	0.0076 (18)	−0.0050 (13)
C26	0.066 (2)	0.0655 (17)	0.078 (2)	−0.0011 (15)	0.0018 (18)	−0.0082 (15)
C27	0.068 (2)	0.0765 (18)	0.0561 (19)	−0.0220 (18)	0.0075 (17)	−0.0103 (15)
C28	0.074 (2)	0.0588 (16)	0.064 (2)	−0.0177 (16)	0.0077 (17)	−0.0007 (13)
C29	0.070 (2)	0.0498 (15)	0.0624 (19)	−0.0120 (13)	0.0097 (16)	−0.0049 (13)
C30	0.084 (3)	0.111 (2)	0.102 (3)	−0.0272 (19)	0.022 (2)	−0.0060 (17)

### Geometric parameters (Å, °)

**Table:**

O1—C8	1.229 (2)	O2—C23	1.233 (2)
N1—C8	1.336 (3)	N2—C23	1.347 (3)
N1—C1	1.432 (3)	N2—C16	1.423 (3)
N1—H1A	0.8596	N2—H2A	0.8594
C1—C6	1.374 (4)	C16—C21	1.368 (4)
C1—C2	1.383 (3)	C16—C17	1.378 (3)
C2—C3	1.385 (4)	C17—C18	1.389 (4)
C2—C7	1.506 (4)	C17—C22	1.511 (4)
C3—C4	1.367 (4)	C18—C19	1.354 (4)
C3—H3A	0.9300	C18—H18A	0.9300
C4—C5	1.367 (4)	C19—C20	1.369 (4)
C4—H4A	0.9300	C19—H19A	0.9300
C5—C6	1.376 (4)	C20—C21	1.385 (4)
C5—H5A	0.9300	C20—H20A	0.9300
C6—H6A	0.9300	C21—H21A	0.9300
C7—H7A	0.9600	C22—H22A	0.9600
C7—H7B	0.9600	C22—H22B	0.9600
C7—H7C	0.9600	C22—H22C	0.9600
C8—C9	1.488 (3)	C23—C24	1.477 (3)
C9—C10	1.387 (3)	C24—C25	1.379 (3)
C9—C14	1.391 (3)	C24—C29	1.380 (3)
C10—C11	1.372 (3)	C25—C26	1.373 (3)
C10—H10A	0.9300	C25—H25A	0.9300
C11—C12	1.378 (3)	C26—C27	1.384 (3)
C11—H11A	0.9300	C26—H26A	0.9300
C12—C13	1.375 (3)	C27—C28	1.376 (3)
C12—C15	1.520 (4)	C27—C30	1.515 (3)
C13—C14	1.362 (3)	C28—C29	1.378 (3)
C13—H13A	0.9300	C28—H28A	0.9300
C14—H14A	0.9300	C29—H29A	0.9300
C15—H15A	0.9600	C30—H30A	0.9600
C15—H15B	0.9600	C30—H30B	0.9600
C15—H15C	0.9600	C30—H30C	0.9600
C15—H15D	0.9887	C30—H30D	0.9501
C15—H15E	0.9457	C30—H30E	1.0098
C15—H15F	0.9744	C30—H30F	0.9793
			
C8—N1—C1	124.69 (19)	C23—N2—C16	123.84 (18)
C8—N1—H1A	117.6	C23—N2—H2A	117.9
C1—N1—H1A	117.7	C16—N2—H2A	118.3
C6—C1—C2	120.9 (3)	C21—C16—C17	119.8 (3)
C6—C1—N1	120.3 (3)	C21—C16—N2	119.3 (3)
C2—C1—N1	118.7 (3)	C17—C16—N2	120.7 (3)
C1—C2—C3	117.7 (3)	C16—C17—C18	118.6 (3)
C1—C2—C7	121.3 (3)	C16—C17—C22	121.2 (3)
C3—C2—C7	121.0 (3)	C18—C17—C22	120.2 (3)
C4—C3—C2	121.9 (3)	C19—C18—C17	121.2 (3)
C4—C3—H3A	119.0	C19—C18—H18A	119.4
C2—C3—H3A	119.0	C17—C18—H18A	119.4
C3—C4—C5	119.3 (3)	C18—C19—C20	120.5 (3)
C3—C4—H4A	120.3	C18—C19—H19A	119.7
C5—C4—H4A	120.3	C20—C19—H19A	119.7
C4—C5—C6	120.3 (3)	C19—C20—C21	118.8 (3)
C4—C5—H5A	119.8	C19—C20—H20A	120.6
C6—C5—H5A	119.8	C21—C20—H20A	120.6
C1—C6—C5	119.9 (3)	C16—C21—C20	121.1 (3)
C1—C6—H6A	120.1	C16—C21—H21A	119.5
C5—C6—H6A	120.1	C20—C21—H21A	119.5
C2—C7—H7A	109.5	C17—C22—H22A	109.5
C2—C7—H7B	109.5	C17—C22—H22B	109.5
H7A—C7—H7B	109.5	H22A—C22—H22B	109.5
C2—C7—H7C	109.5	C17—C22—H22C	109.5
H7A—C7—H7C	109.5	H22A—C22—H22C	109.5
H7B—C7—H7C	109.5	H22B—C22—H22C	109.5
O1—C8—N1	120.8 (2)	O2—C23—N2	120.2 (2)
O1—C8—C9	120.7 (2)	O2—C23—C24	122.3 (2)
N1—C8—C9	118.4 (2)	N2—C23—C24	117.5 (2)
C10—C9—C14	116.8 (3)	C25—C24—C29	118.6 (2)
C10—C9—C8	124.5 (2)	C25—C24—C23	118.6 (2)
C14—C9—C8	118.5 (2)	C29—C24—C23	122.8 (2)
C11—C10—C9	120.8 (2)	C26—C25—C24	120.4 (2)
C11—C10—H10A	119.6	C26—C25—H25A	119.8
C9—C10—H10A	119.6	C24—C25—H25A	119.8
C10—C11—C12	121.9 (3)	C25—C26—C27	121.5 (3)
C10—C11—H11A	119.1	C25—C26—H26A	119.3
C12—C11—H11A	119.1	C27—C26—H26A	119.3
C13—C12—C11	117.3 (3)	C28—C27—C26	117.7 (3)
C13—C12—C15	121.2 (3)	C28—C27—C30	120.9 (2)
C11—C12—C15	121.5 (3)	C26—C27—C30	121.4 (3)
C14—C13—C12	121.4 (3)	C27—C28—C29	121.3 (2)
C14—C13—H13A	119.3	C27—C28—H28A	119.4
C12—C13—H13A	119.3	C29—C28—H28A	119.4
C13—C14—C9	121.7 (2)	C28—C29—C24	120.5 (2)
C13—C14—H14A	119.1	C28—C29—H29A	119.7
C9—C14—H14A	119.1	C24—C29—H29A	119.7
C12—C15—H15A	109.5	C27—C30—H30A	109.5
C12—C15—H15B	109.5	C27—C30—H30B	109.5
H15A—C15—H15B	109.5	H30A—C30—H30B	109.5
C12—C15—H15C	109.5	C27—C30—H30C	109.5
H15A—C15—H15C	109.5	H30A—C30—H30C	109.5
H15B—C15—H15C	109.5	H30B—C30—H30C	109.5
C12—C15—H15D	109.4	C27—C30—H30D	115.3
H15A—C15—H15D	54.8	H30A—C30—H30D	58.1
H15B—C15—H15D	57.7	H30B—C30—H30D	52.6
H15C—C15—H15D	141.1	H30C—C30—H30D	135.2
C12—C15—H15E	112.3	C27—C30—H30E	110.2
H15A—C15—H15E	138.2	H30A—C30—H30E	140.3
H15B—C15—H15E	53.8	H30B—C30—H30E	57.9
H15C—C15—H15E	57.7	H30C—C30—H30E	54.3
H15D—C15—H15E	107.7	H30D—C30—H30E	105.2
C12—C15—H15F	110.8	C27—C30—H30F	111.6
H15A—C15—H15F	54.7	H30A—C30—H30F	58.1
H15B—C15—H15F	139.7	H30B—C30—H30F	138.8
H15C—C15—H15F	57.4	H30C—C30—H30F	53.7
H15D—C15—H15F	106.4	H30D—C30—H30F	109.3
H15E—C15—H15F	110.1	H30E—C30—H30F	104.5
			
C8—N1—C1—C6	63.9 (3)	C23—N2—C16—C21	97.6 (3)
C8—N1—C1—C2	−118.2 (3)	C23—N2—C16—C17	−86.8 (3)
C6—C1—C2—C3	0.1 (3)	C21—C16—C17—C18	−0.7 (3)
N1—C1—C2—C3	−177.9 (2)	N2—C16—C17—C18	−176.4 (2)
C6—C1—C2—C7	178.8 (2)	C21—C16—C17—C22	177.1 (2)
N1—C1—C2—C7	0.8 (3)	N2—C16—C17—C22	1.4 (3)
C1—C2—C3—C4	0.4 (4)	C16—C17—C18—C19	0.8 (4)
C7—C2—C3—C4	−178.2 (2)	C22—C17—C18—C19	−177.0 (3)
C2—C3—C4—C5	−0.5 (4)	C17—C18—C19—C20	−0.1 (4)
C3—C4—C5—C6	−0.1 (4)	C18—C19—C20—C21	−0.7 (4)
C2—C1—C6—C5	−0.6 (3)	C17—C16—C21—C20	0.0 (3)
N1—C1—C6—C5	177.3 (2)	N2—C16—C21—C20	175.7 (2)
C4—C5—C6—C1	0.6 (4)	C19—C20—C21—C16	0.8 (4)
C1—N1—C8—O1	−3.2 (4)	C16—N2—C23—O2	−1.5 (4)
C1—N1—C8—C9	174.6 (3)	C16—N2—C23—C24	177.9 (3)
O1—C8—C9—C10	164.4 (3)	O2—C23—C24—C25	28.9 (4)
N1—C8—C9—C10	−13.4 (4)	N2—C23—C24—C25	−150.4 (2)
O1—C8—C9—C14	−10.5 (4)	O2—C23—C24—C29	−152.7 (2)
N1—C8—C9—C14	171.7 (2)	N2—C23—C24—C29	27.9 (4)
C14—C9—C10—C11	1.7 (4)	C29—C24—C25—C26	−1.7 (4)
C8—C9—C10—C11	−173.2 (3)	C23—C24—C25—C26	176.6 (2)
C9—C10—C11—C12	0.3 (4)	C24—C25—C26—C27	1.1 (4)
C10—C11—C12—C13	−2.1 (4)	C25—C26—C27—C28	0.8 (4)
C10—C11—C12—C15	176.0 (3)	C25—C26—C27—C30	−179.3 (3)
C11—C12—C13—C14	1.9 (4)	C26—C27—C28—C29	−2.0 (4)
C15—C12—C13—C14	−176.2 (3)	C30—C27—C28—C29	178.0 (2)
C12—C13—C14—C9	0.1 (4)	C27—C28—C29—C24	1.4 (4)
C10—C9—C14—C13	−2.0 (4)	C25—C24—C29—C28	0.5 (4)
C8—C9—C14—C13	173.3 (3)	C23—C24—C29—C28	−177.8 (2)

### Hydrogen-bond geometry (Å, °)

**Table:**

*D*—H···*A*	*D*—H	H···*A*	*D*···*A*	*D*—H···*A*
N1—H1A···O2	0.86	2.05	2.878 (2)	163
N2—H2A···O1^i^	0.86	2.05	2.883 (2)	162

Symmetry codes: (i) *x*, *y*−1, *z*.
